# Patterns of disease presentation and management in Egyptian primary care: findings from a survey of 2458 primary care patient consultations

**DOI:** 10.1186/1471-2296-14-161

**Published:** 2013-10-22

**Authors:** Ahmed Aboulghate, Gary Abel, Georgios Lyratzopoulos, Aida Abdelmohsen, Ahmed R Hamed, Martin Roland

**Affiliations:** 1 Institute of Public Health, Cambridge Centre for Health Services Research, Robinson way Forvie site, Cambridge CB2 0SR, UK; 2 National Research Centre, Egypt, Behooth street, Giza, Egypt; 3 The Management Centre, American University in Cairo, PO Box 2511, Cairo, Egypt

**Keywords:** Family practice, Primary health care, Egypt, Morbidity, Epidemiology, Demography, Physician’s practice patterns, Health care surveys, Health services research, Health facility administration

## Abstract

**Background:**

The Egyptian government is considering embarking on a new wave of health sector reform. Although primary care is seen as central to the anticipated reforms, little is known about the current morbidity and utilization patterns in Egyptian publicly funded primary care. We conducted this survey study of patient encounters to describe the demographic characteristics of patients attending publicly-funded primary care practices, the relative frequency of conditions encountered in these practices, and the rates of drug prescription, investigation and referral.

**Method:**

Cross-sectional survey of twelve primary care practices and 2458 patient consultations. Additional secondary data were collected from five of the twelve practices for preventive services provided at these practices i.e. immunizations, family planning and ante-natal care.

**Results:**

54% of the attendances were for people below the age of twenty, of which 54% were females. In patients above the age of twenty, women accounted for 73% of consultations. Upper respiratory tract infection was the most common reason for encounter, accounting for 24% of the presentations, followed by gastroenteritis (10%), intestinal parasites (5%), and lower respiratory tract infections (5%). Over 97% of patients were prescribed at least one drug, whereas investigation and referral rates were low (15% and 5% respectively). When the analysis was repeated for practices where data on both curative and preventive services were available (5 practices and 2146 consultations), substantial proportions of patients were found to seek care for immunizations (25%), family planning (12%), and ante-natal care (11%).

**Conclusion:**

Most patients utilizing primary care practices in Egypt seek care for minor and preventive services with relatively few consultations for more serious conditions. There is also a pattern of prescribing drugs to most primary care patients which may reflect over-prescribing by primary care doctors.

## Background

Over recent decades, developing as well as developed countries have undergone iterative cycles of reform in their healthcare sectors. Across many different local contexts, the generic goals of health reforms have considerable similarity and include improving healthcare quality, containing cost and enhancing equity. In many of these reforms, primary care has been central to achieving these three objectives [1]. Egypt initiated its long-term Health Sector Reform Project in 1997. At the core of the reform was the country’s primary care system [2]. The Egyptian government invested substantially aiming to shift the focus of healthcare from reliance on specialist care to a less costly and more widely accessible primary care based model. Today around 5000 public primary care practices run by the Ministry of Health (MOH) serve the 83 million Egyptians and are geographically and financially accessible to almost all the population [3]. However, after 15 years of the reform these practices are still widely perceived by the public as poor quality care provider, and this is reflected in underutilization of these practices. Only 6% of patients seeking outpatient care choose MOH primary care practices, whereas 14% self-refer themselves to public hospitals and secondary care providers, and around 80% choose private, fee-for-service providers [4,5]. Following the major political shifts in the aftermath of Egypt’s “January 25th Revolution” in 2011, the newly appointed administration is considering embarking on a new wave of health sector reform. Although primary care is seen as central to the anticipated reforms, little is known about the current morbidity and utilization patterns in Egyptian primary care. Such information is critical to inform decision making about potential reforms to improve the quality of care provision in these services [6]. In light of these observations, we conducted this survey of patient encounters to describe the demographic characteristics of patients attending MOH primary care practices, the relative frequency of conditions encountered there, and the frequency of drug prescription, investigation and referral.

## Methods

### Sample

Egypt is divided into 27 governorates classified as one of: urban, frontier (desert), upper Egypt or lower Egypt governorate. There are five urban governorates, while the rest cover urban and rural areas (57% of the population live in rural areas). There are 5,136 MOH primary care practices in Egypt, 81% of which lie in rural areas [7]. A purposive sample of twelve practices was chosen taking account of three attributes of practices and their location. These attributes were: 1) the rurality of the practice catchment area (rural/urban); 2) the accreditation status of the practice (41% of practices are accredited through a national accreditation scheme) [8]; and 3) the category of governorate (due to time and resources constraint, frontier governorates were not included in our sample). Twelve groups of practices were identified to represent all combinations of the three variables. One practice from each group was then chosen according to convenience.

The sample size was calculated to achieve a precision (95% CI) of +/− 1% for a condition with 5% prevalence, assuming an intraclass correlation (ICC) of 0.015 [9]. The minimum sample size was estimated to be 207 patient consultations per practice, i.e. a total of 2484 consultations. The study had full ethical approval from Cambridge Psychology Research Ethics Committee (No. 2011.34).

### Data

Data collection forms were designed to collect information on patients’ demographic characteristics, presenting complaints, initial diagnoses and whether a prescription, referral, or an investigation was ordered during their visit. The forms were designed to be completed by primary care doctors during or after the consultation with patients. Up to three diagnoses were allowed to be recorded for each patient, each of which was coded as a separate consultation. Most doctors collected the data in English since medicine in Egypt is taught in English. Data collected in Arabic were translated into English prior to assigning a reason for the encounter which was then assigned for all consultations using the ‘International Classification of Primary Care’ (ICPC) version 4.1. The ICPC is an international classification developed by the World Health Organization based on the International Classification of Diseases (ICD). ICPC codes reasons for encounter rather than specific diseases or diagnoses and assigns codes to different complaints, diagnoses, as well as curative, preventive and diagnostic services provided at the primary care level. The diagnosis reported by the doctor was reported as the reason for encounter for all patients. Patients for whom no definite diagnosis was reached, the main presenting complaint was used as the reason for encounter. Codes that represent the same medical condition were later aggregated into a unifying common condition.

Immunizations, family planning and antenatal services are also provided at the primary care practices but are administered separately. These services will be referred to hereafter as ‘preventive’ services while services for all other conditions will be referred to as ‘curative’ services. For curative care, primary data were collected from all participating practices. For preventive conditions, data were collected from five of the twelve participating practices using practice medical records. Demographic and management data could not be identified for the preventive encounters.

### Analysis

The analysis was performed using data collected from the twelve practices on curative care. Descriptive analyses were conducted on the patients’ socio-demographic characteristics, reasons for the primary care encounter and frequency of prescription, investigation and referral.

In order to make our results more representative of all visits to MOH primary care practices we applied a weighting depending on the rurality of practices. The weights accounted for the fact that rural practices were under-represented in our sample, and the fact that primary care encounters in MOH practices are more common in rural areas. Details of how the weights were derived are given in Appendix A.

To compare the frequency of presentation of curative and preventive conditions, we finally repeated the analysis including consultations for preventive services after restricting the analysis to the five practices for which these data were available. It will be noted in the results whenever the analysis was restricted to this group of practices. SPSS v.18 was used for data analysis.

## Results

After excluding patients with missing data, information was available for 2396 patient visits resulting in 2458 diagnoses. Four of the twelve practices did not meet the target of 207 patient encounters, collecting data for 186, 179, 158 and 113 consultations only. The sample sizes in the other practices ranged from 209 to 260 patient consultations. The overall mean of patient encounters collected was 205 consultations/practice.

**Figure 1:**
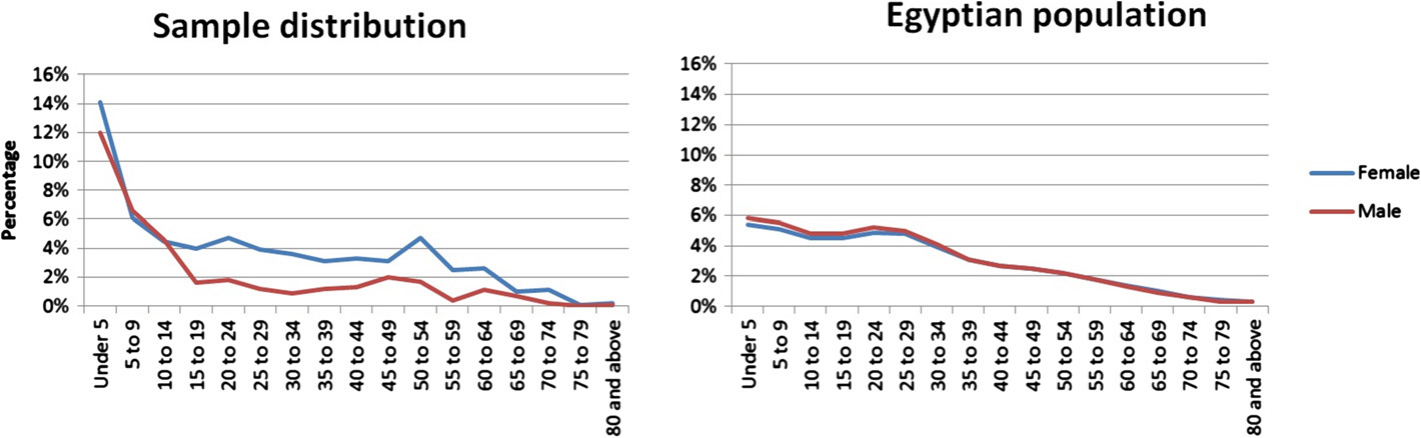
Age and sex distributions of the patient sample compared to Egyptian population.

Analysis of the socio-demographic characteristics of patients reveals different utilization across gender and age groups with women and younger patients utilizing primary care services substantially more than men and older patients. Patients below twenty years of age accounted for more than half of the consultations (54%). The number of consultations decreased as age increased, reaching 0.2% in the patient group aged 80 or above. Females accounted for 63% of all encounters. This gender difference was more evident in patients older than twenty years of age (73% of consultations were for females, compared to 54% in those under the age of twenty). To identify whether this difference was due to more incidence of obstetric and gynaecological conditions compared to andrological ones, we repeated the analysis excluding gynaecological, obstetric and andrological conditions. Females still accounted for more consultations (62%). The age and sex distributions of patient encounters and the Egyptian population are presented in Table 1 and Figure 1.

**Table 1:** Age and gender distribution* of patient encounters and Egyptian population**

	**Male**		**Female**		**Total**	
	**Sample**	**Population**	**Sample**	**Population**	**Sample**	**Population**
**Under 5**	294 (12%)	4861 (5.8%)	346 (14.1%)	4481 (5.4%)	640 (26.2%)	9342 (11.2%)
**5-9**	162 (6.6%)	4610 (5.5%)	149 (6.1%)	4259 (5.1%)	311 (12.7%)	8869 (10.6%)
**10-14**	112 (4.6%)	4038 (4.8%)	111 (4.5%)	3765 (4.5%)	223 (9.1%)	7803 (9.3%)
**15-19**	40 (1.6%)	4038 (4.8%)	97 (4%)	3805 (4.5%)	137 (5.6%)	7843 (9.4%)
**20-24**	45 (1.8%)	4309 (5.2%)	115 (4.7%)	4089 (4.9%)	160 (6.5%)	8398 (10%)
**25-29**	30 (1.2%)	4152 (5%)	96 (3.9%)	3987 (4.8%)	126 (5.1%)	8139 (9.7%)
**30-34**	21 (0.9%)	3403 (4.1%)	88 (3.6%)	3300 (3.9%)	109 (4.5%)	6703 (8%)
**35-39**	30 (1.2%)	2633 (3.1%)	76 (3.1%)	2564 (3.1%)	106 (4.3%)	5197 (6.2%)
**40-44**	31 (1.3%)	2300 (2.7%)	81 (3.3%)	2241 (2.7%)	112 (4.6%)	4541 (5.4%)
**45-49**	48 (2%)	2113 (2.5%)	75 (3.1%)	2069 (2.5%)	123 (5%)	4182 (5%)
**50-54**	41 (1.7%)	1852 (2.2%)	114 (4.7%)	1827 (2.2%)	155 (6.3%)	3679 (4.4%)
**55-59**	10 (0.4%)	1519 (1.8%)	62 (2.5%)	1504 (1.8%)	72 (2.9%)	3023 (3.6%)
**60-64**	27 (1.1%)	1124 (1.3%)	64 (2.6%)	1130 (1.4%)	91 (3.7%)	2254 (2.7%)
**65-69**	16 (0.7%)	781 (0.9%)	25 (1%)	797 (1%)	41 (1.7%)	1578 (1.9%)
**70-74**	6 (0.2%)	510 (0.6%)	27 (1.1%)	525 (0.6%)	33 (1.3%)	1035 (1.2%)
**75-80**	0 (0%)	291 (0.3%)	2 (0.1%)	303 (0.4%)	2 (0.1%)	594 (0.7%)
**80 & older**	2 (0.1%)	239 (0.3%)	4 (0.2%)	242 (0.3%)	6 (0.2%)	481 (0.6%)
**Total**	915	42773	1532	40888	2447	8366
	(37.1%)	(51.1%)	(62.9%)	(48.9%)	(100%)	(100%)

*Reported percentages are of total consultations/population.

**2012 population estimates. Population figures shown in 1000 s.

Analysis of the reasons for encountering care by ICPC chapters reveals highest consultation rates for respiratory conditions (34%) followed by digestive (20%), skin (12%) and circulatory conditions (Table 2). Looking at the conditions in more detail, the commonest presenting condition was upper respiratory tract infection (URTI) accounting for more than 1 in every 5 encounters (24%). The second most common condition was gastroenteritis (9.5%) followed by intestinal parasites (5.5%), lower respiratory tract infection (5.4%), skin infections (4.8%), non-infective dermatitis (4.3%), anaemia (4.3%) and hypertension (3.9%). The frequency and percentage of presentation of the commonest 30 conditions are presented in Table 3.

**Table 2:** Reasons for encountering care for curative conditions by ICPC chapters (12 practices, 2458 consultations)

**Chapter**	**Consultations**		Weighted percentage*
	**Frequency**	**Percentage**	
**Respiratory**	783	31.9	34.2
**Digestive**	477	19.4	20.4
**Skin**	268	10.9	12.4
**Circulatory**	220	9.0	5.8
**Eye**	83	3.4	4.6
**Blood/Lymphatics**	114	4.6	4.6
**Urology**	74	3.0	3.7
**Endocrine**	126	5.1	3.5
**Musculoskeletal**	101	4.1	3.4
**General/Unspecified**	72	2.9	3.2
**Gynaecological**	62	2.5	1.8
**Ear**	64	2.6	1.7
**Obstetric**	5	0.2	0.3
**Andrology**	2	0.1	0.2
**Neurological**	7	0.3	0.2
**Psychological**	0	0	0
**Social**	0	0	0

*Weighted for different population and utilisation rates between urban and rural areas.

**Table 3:** Presentation of the 30 most common conditions (12 practices, 2458 consultations)

**Condition**	**Consultations**		**Weighted**	**Rural**		**Urban**		p- value**
	**n**	**%**	percentage*	**n**	**%**	**n**	**%**	
Upper Respiratory Tract Infection	537	21.8	24.0	227	25.0	310	20.0	0.04
Gastroenteritis	227	9.2	9.5	87	9.6	140	9.0	0.66
Intestinal parasites	109	4.4	5.5	54	5.9	55	3.6	0.01
Lower Respiratory Tract Infection	128	5.2	5.4	50	5.5	78	5.0	0.61
Skin infection	102	4.1	4.8	46	5.1	56	3.6	0.08
Dermatitis	107	4.4	4.3	39	4.3	68	4.4	0.91
Anaemia	108	4.4	4.3	38	4.2	70	4.5	0.69
Hypertension	161	6.6	3.9	24	2.6	137	8.8	<0.005
Conjunctivitis	71	2.9	3.9	39	4.3	32	2.1	<0.005
Asthma	55	2.2	2.7	27	3.0	28	1.8	0.06
Urinary Tract Infection	46	1.9	2.7	28	3.1	18	1.2	<0.005
Diabetes	96	3.9	2.7	19	2.1	77	5.0	<0.005
Chickenpox	21	0.9	1.7	19	2.1	2	0.1	<0.005
Ear infection	63	2.6	1.6	11	1.2	52	3.4	<0.005
Irritable Bowel Syndrome	37	1.5	1.1	8	0.9	29	1.9	0.05
Osteoarthrosis	48	2.0	1.0	5	0.6	43	2.8	<0.005
Postural hypotension	26	1.1	0.7	4	0.4	22	1.4	0.02
Rheumatic fever/heart disease	24	1.0	0.7	5	0.6	19	1.2	0.1
Musculoskeletal disease-other	9	0.4	0.6	7	0.8	2	0.1	0.01
Throat symptom/ complaint	34	1.4	0.6	2	0.2	32	2.1	<0.005
Abnormal urine test, non-specified	9	0.4	0.6	6	0.7	3	0.2	0.07
Mouth/tongue/lip disease	14	0.6	0.6	5	0.6	9	0.6	0.92
Back syndrome/ pain	12	0.5	0.5	5	0.6	7	0.5	0.74
Perinatal morbidity- other	11	0.4	0.5	5	0.6	6	0.4	0.56
Oesophageal disease/ulcer	7	0.3	0.5	5	0.6	2	0.1	0.06
Dyspepsia/ indigestion	6	0.2	0.5	5	0.6	1	0.1	0.02
Skin burn/scald	6	0.2	0.5	5	0.6	1	0.1	0.02
Nutritional deficiency	23	0.9	0.4	2	0.2	21	1.4	0.01
Female genital infection	17	0.7	0.4	3	0.3	14	0.9	0.1
Liver disease- non-specified	5	0.2	0.4	5	0.6	0	0	0.00

*Weighted for different population and utilisation rates between urban and rural areas.

**Chi-squared test for differences in prevalence between urban/rural practices.

When analysis was restricted to practices for which data on both curative and preventive services were available, preventive care was found to be a major reason for encountering primary care. The three preventive conditions; immunisations, family planning and antenatal care accounted for more than 40% of all visits. Immunization visits comprised 24.9%, family planning 11.6% and antenatal care 10.8% (Table 4).

**Table 4:** Presentation of most common preventive and curative conditions (2146 encounters in five practices where data was available for both preventive and curative care)

**Condition**	**Consultations**		**Weighted**	**Rural**		**Urban**		p-value**
	**n**	**%**	percentage*	**n**	**%**	**n**	**%**	
Immunization	534	24.9	24.9	99	22.9	435	25.4	0.29
Upper Respiratory Tract Infection	289	13.5	13.5	62	14.4	227	13.2	0.55
Family planning	248	11.6	11.6	47	10.9	201	11.7	0.62
Ante-natal care	232	10.8	10.8	63	14.6	169	9.9	0.01
Gastroenteritis	134	6.2	6.2	47	10.9	87	5.1	<0.005
Intestinal parasites	68	3.2	3.2	14	3.2	54	3.2	0.92
Skin infections	57	2.7	2.7	11	2.5	46	2.7	0.87
Lower Respiratory Tract Infection	55	2.6	2.6	5	1.2	50	2.9	0.04
Conjunctivitis	54	2.5	2.5	15	3.5	39	2.3	0.16
Anaemia	49	2.3	2.3	11	2.5	38	2.2	0.68
Dermatitis	48	2.2	2.2	9	2.1	39	2.3	0.81
Urinary Tract Infection	31	1.4	1.5	3	0.7	28	1.6	0.14
Asthma	27	1.3	1.3	0	0	27	1.6	0.01
Hypertension	25	1.2	1.2	1	0.2	24	1.4	0.04
Chickenpox	19	0.9	0.9	0	0	19	1.1	0.03
Diabetes	19	0.9	0.9	0	0	19	1.1	0.03
Ear infection	14	0.7	0.7	3	0.7	11	0.6	0.9

*Weighted for different population and utilisation rates between urban and rural areas.

**Chi-squared test for differences in prevalence between urban/rural practices.

Analysis of the prescription, investigation and referral rates for curative conditions revealed high rates of drug prescription. In 97.1% of patient encounters the doctor prescribed some form of medication to the patient. Across practices, the prescription rates were consistently high ranging from 93% to 100%. Both investigation and referral rates were relatively lower, investigation rates ranged from 0% to 26% (mean 15.1%), while referral rates ranged from 0% to 20% (mean 4.9%).

## Discussion

### Overview of the main findings

The utilization of primary care services varied across different socio-demographic patient groups in our sample. Women were more frequent users of primary care services. This difference was most evident in age groups 20–59 and was minimal in extremes of age. In our sample this could be explained only in part by the frequency of presentation of gynaecological and obstetric conditions compared to andrological conditions. When the analysis was repeated excluding these conditions, women still accounted for more primary care visits than men. This suggests higher utilization by women, independent from the incidence of gender-dependant conditions, which is observed in several other countries [10,11]. Young patients also made substantially more visits to primary care practices compared to older ones. More than a quarter of the patients in our study were under the age of five, with frequency of consultation decreasing as age increases. Since our study reports frequency of consultations and not the utilization rates (visits/person/year), it is likely that the observed distribution is –at least in part- attributed to the age distribution of the Egyptian population which is skewed towards younger demographics [12]. It should be noted, however, that our results are not adjusted for the socio-demographic make-up of the practices catchment populations or how they resemble the make-up of the Egyptian population.

The majority of patients seeking curative services at primary care practices were found to present with relatively minor conditions (as respiratory and gastrointestinal infections) rather than more serious ones. Even serious conditions which are estimated to be highly prevalent in Egypt, such as liver cirrhosis, ischaemic heart disease, neuropsychiatric conditions and various types of cancers [13] were rarely encountered in our sample. This finding could be explained by the absence of a gate-keeping mechanism in the Egyptian healthcare system, so these patients often self-refer themselves to specialist or private care. Moreover, the high presentation of infectious conditions may suggest that the prevalence of communicable diseases in Egypt is still high and that Egypt as many other developing countries is facing a dual burden of disease; a persistently high burden of communicable diseases and a rapidly increasing burden of non-communicable diseases [14]. It was further observed that infectious conditions present more commonly in rural practices, whereas chronic conditions were more common in urban practices. This potentially reflects the less sanitary conditions in the more deprived rural areas. Also the higher presentation of conditions like hypertension and diabetes in urban areas is likely to be attributable to the metabolic risk factors related to the life-style in urban areas.

Notably, while neuropsychiatric conditions account for 15% of the national burden of disease [13], none of the 2458 consultations was reported to be for a psychiatric condition. Beside self-referral to specialist care, this could be attributed to a number of factors. There is considerable social and cultural stigma, taboos and misconceptions attached to mental disorders in Egypt which may discourage neuropsychiatric patients from seeking medical care for their conditions [15]. Alternatively, patients with psychiatric conditions may present with physical symptoms (i.e. somatisation) and may be misdiagnosed by the doctor as having physical condition. In addition, traditional/spiritual healers are still consulted by some Egyptians particularly for psychiatric conditions [15].

A substantial proportion of those attending primary care practices were found to be seeking preventive services, i.e. antenatal care, family planning and particularly immunizations. This could be attributed to the central support and high political priority given to these three services. Also, unlike curative care, there are limited numbers of private providers of immunization services and they are relatively expensive and geographically scattered. Immunization is perceived by the public to be a simple procedure that primary care providers can provide effectively and consequently does not require seeking the more expensive, and less accessible private care.

High rates of drug prescription were reported, whereas the observed investigation and referral rates were relatively low. The high prescribing rate by primary care doctors has been acknowledged as a challenge by the Ministry of Health [5], and previous efforts have attempted to change primary care prescribing behaviour. The findings of this study suggest, however, that over-prescribing may still be prevalent in Egyptian primary care and should be further investigated, including whether similar patterns are observed in secondary and tertiary care.

### Comparison with existing literature

The only study identified on patterns of morbidity in Egyptian primary care was an unpublished study conducted in 2001 for the Health Sector Reform Project [16]. The study results were included in a technical report aiming to evaluate the services provision in primary care practices to guide the reform process. Data were collected from a sample of around 28,300 patients seeking care at four primary care practices over a period of six months. The conditions were coded using ICD-10 and only the ten most common conditions were reported. While the outcomes of this study could not be directly compared to ours due to different coding and aggregation techniques, some findings may still be inferred from a comparison of the two. Conditions as respiratory infections, hypertension and anaemia have been and still are commonly seen in primary care. Gastrointestinal infection was not reported among the most common ten conditions in this study. This could be due to the high specificity of ICD-10 codes and consequently patients with gastro-intestinal infections were categorized under multiple smaller groups by factors such as the infective organism, presenting symptom or site of infection. The most evident difference between the studies – and the most difficult to account for - is the high presentation of arthritis in this study (9%) compared to our findings where arthritis accounted for only 1% of consultations.

Another study was identified which addressed the reasons for healthcare encounters in Egypt which included all levels of care, not just the primary care level [4]. A sample of 12,002 Egyptian households, covering 56,305 individuals was surveyed addressing the reasons for encountering care in any healthcare facility within the previous four weeks. Again, this study concluded that vaccination, antenatal care, family planning, respiratory infections, gastroenteritis and hypertension were among the most common reasons for encountering healthcare. However, beside these conditions, more serious ones as hepatitis, typhoid, rheumatic, renal and cardiac conditions were commonly reported. This finding supports our interpretation of the relative under-representation of serious conditions being because these patients self-refer themselves to specialist care.

Many of our results are consistent with those from other countries. The higher utilization of healthcare services by females and patients at extremes of age is almost universal [11,17-23]. Also, minor and infectious conditions have been found to constitute a high proportion of primary care consultations in many countries, particularly developing ones where infectious diseases are common. Similar studies from many developing countries present similar patterns of disease presentation with relative infrequency of chronic and severe conditions [19-23]. However, the proportion of patients seeking care for preventive conditions is substantially higher in our study than in other studies. This is potentially explained by differences in the availability and accessibility of other providers for preventive services across different countries.

### Strengths and limitations

To the best of the authors’ knowledge, this is the only published study describing primary care utilization in Egypt in detail. The data were collected from a sample of practices providing variation in the main factors that are likely to affect the patterns of disease presentation and management in Egypt. There is, however, a possibility that data collected for certain conditions were less comprehensive than for others, which would introduce bias. For example, consultations for more complex and serious conditions might have been lengthier and more labour intensive leaving doctors after such consultations in a hurry to compensate for the time lost and potentially failing to document subsequent consultations. There is also a possibility that data on preventive conditions were more comprehensive than data for curative ones. This is because data for curative conditions were collected voluntarily by participants in our data collection forms, while data on preventive conditions were collected from practice records which could potentially be given higher priority for data completeness. To account for this, participants were deliberately asked to collect the reasons for encountering care for all patients, continuously, until the target sample size was reached. The risk of selective exclusion of patients was highlighted to participants. Upon recruitment, the author further supervised participants as they collected data for a sample of patients to ensure the data collection process was accurate and to address any ambiguities.

Also any inconsistency in identifying the labels describing the reason for encountering care could affect the reported results. The reason for encounter in our study was assigned centrally based on the patient’s complaint and the diagnosis given by the doctor who saw the patient. The data were coded centrally by the same researcher (AA) to avoid inconsistencies and to minimize errors at this stage.

Seasonal variations of disease pattern were not accounted for in this study. Data collection occurred over the months of July and August and caution should be taken if this study is used to interpret the annual disease presentation rates. It is likely that conditions which are more common in winter (e.g. respiratory infections) are under-represented in this study whereas summer conditions (e.g. gastrointestinal infections) may have been overrepresented. In order to estimate the size of the seasonal variation effect, we compared our results to those from the healthcare utilization household survey (presented above) [4], which was conducted in two phases, summer and winter. As anticipated, respiratory infections were less common in our study (22% compared to 35%) whereas gastroenteritis was found to be more common (9% vs. 5%). We thus acknowledge that respiratory infections may be under-represented and gastrointestinal infections may be over-represented compared to annual presentation rates.

### Research recommendations

This study provides an illustration of patterns of disease presentation and sheds light on the substantial scope for future research in the Egyptian primary care. The study used a purposive sample of twelve primary care practices from three different governorates. A random sample of practices from all twenty seven governorates of Egypt would give more generalizable results and allow for identifying differences in disease presentation –and accordingly differences in health needs- in different geographical areas across Egypt.

Future reform efforts aiming to foster the role of primary care need a clearer understanding as to why patients refrain from seeking care at primary care practices for conditions that could be addressed at that level– particularly mental health conditions. Qualitative studies with households or patients seeking care could be used to identify the underlying reasons why people do not attend primary care practices, and how the public trust in the services could, if appropriate, be restored.

The rate of prescribing observed in primary care is an observation that merits further research. Future studies should look into the numbers and types of drugs prescribed for each presentation to investigate whether an over-prescribing behaviour is prevalent among primary care doctors. Such studies could include prescribing in secondary and tertiary care settings and how this affects national healthcare expenditure.

## Conclusion

In conclusion, this survey has revealed the content and morbidity profile of Egyptian primary care and shed light on priority areas for future health services research. The majority of patients attending primary care practices are seeking care for preventive and minor conditions. It is important to understand why some patients still refrain from seeking care at primary care practices for conditions which are managed at this level of care in other countries. It is also important to investigate the appropriateness of drug prescribing by primary care doctors and to explore whether an overprescribing behavior is prevalent at this or other levels of care. These findings will inform policy makers in the anticipated new cycle of health reform and can aid effective allocation of resources and services based on local health needs.

### Appendix A – Derivation of weights

Our sample consisted of more patient visits to practices in urban areas (1549, 65%) than in rural areas (909, 35%). In contrast the number of total practice visits is thought to be higher in rural areas. Firstly 57% of the Egyptian population live in rural areas [7] and whilst they make slightly fewer visits to outpatient facilities of any kind (9.19 per person per years rural compared to 9.99 per person per year in urban areas) [4], the percentage of outpatient visits occurring at MOH practices is considerably higher (8.3% vs. 2.5%) [4]. Further as there is evidence that the distribution of presenting conditions vary by rurality, it is important to reflect this imbalance between sample and reality by using appropriate weighting based on these figures. The weight, W_*i*_, for each observation, *i*, are given as by:

(A1) $$W_{i}\;=\;N\;\frac{r_{i}\;v_{i\;}m_{i}}{n_{i}}\;\frac{1}{\sum_{i=0}^{N}\frac{n_{i}}{r_{i\;}v_{i}\;m_{i}}}$$

Where *r*_*i*_ is the proportion of the Egyptian population living in the same rurality category as observation *i*, *v*_*i*_ is the mean number of visits to outpatient facilities of any kind for residents in the same rurality category as observation *i*, and *m*_i_ is the proportion of outpatient visits occurring at MOH practices in the same rurality category as observation *i*.

## Abbreviations

ICPC: International classification of primary care; ICD: International classification of diseases; MOH: Ministry of Health.

## Competing interests

The authors declare that they have no competing interests.

## Authors’ contributions

All authors read and approved the final manuscript.

## Authors’ information

This work relates to part-fulfilment (dissertation) of the PhD degree in Public Health from of the Department of Public Health and Primary Care, University of Cambridge, by AA, who is a beneficiary of the Open Society Foundation scholarship through the Cambridge Overseas Trust. GL is supported by a post-doctoral fellowship award by the National Institute for Health Research (NIHR PDF-2011-04-047). The views expressed in this publication are those of the author and not necessarily those of the NHS, the National Institute for Health Research, or the Department of Health.

## Pre-publication history

The pre-publication history for this paper can be accessed here:

http://www.biomedcentral.com/1471-2296/14/161/prepub
